# Development of a Multisensory Wearable System for Monitoring Cigarette Smoking Behavior in Free-Living Conditions

**DOI:** 10.3390/electronics6040104

**Published:** 2017-11-28

**Authors:** Masudul Haider Imtiaz, Raul I. Ramos-Garcia, Volkan Yusuf Senyurek, Stephen Tiffany, Edward Sazonov

**Affiliations:** 1 Department of Electrical and Computer Engineering, The University of Alabama, Tuscaloosa, AL 35487, USA; 2 Department of Psychology, University at Buffalo, The State University of New York, Buffalo, NY 14260, USA

**Keywords:** accelerometer, bio-impedance, cigarette smoking, ECG, GPS, PACT, RIP

## Abstract

This paper presents the development and validation of a novel multi-sensory wearable system (Personal Automatic Cigarette Tracker v2 or PACT2.0) for monitoring of cigarette smoking in free-living conditions. The contributions of the PACT2.0 system are: (1) the implementation of a complete sensor suite for monitoring of all major behavioral manifestations of cigarette smoking (lighting events, hand-to-mouth gestures, and smoke inhalations); (2) a miniaturization of the sensor hardware to enable its applicability in naturalistic settings; and (3) an introduction of new sensor modalities that may provide additional insight into smoking behavior e.g., Global Positioning System (GPS), pedometer and Electrocardiogram(ECG) or provide an easy-to-use alternative (e.g., bio-impedance respiration sensor) to traditional sensors. PACT2.0 consists of three custom-built devices: an instrumented lighter, a hand module, and a chest module. The instrumented lighter is capable of recording the time and duration of all lighting events. The hand module integrates Inertial Measurement Unit (IMU) and a Radio Frequency (RF) transmitter to track the hand-to-mouth gestures. The module also operates as a pedometer. The chest module monitors the breathing (smoke inhalation) patterns (inductive and bio-impedance respiratory sensors), cardiac activity (ECG sensor), chest movement (three-axis accelerometer), hand-to-mouth proximity (RF receiver), and captures the geo-position of the subject (GPS receiver). The accuracy of PACT2.0 sensors was evaluated in bench tests and laboratory experiments. Use of PACT2.0 for data collection in the community was validated in a 24 h study on 40 smokers. Of 943 h of recorded data, 98.6% of the data was found usable for computer analysis. The recorded information included 549 lighting events, 522/504 consumed cigarettes (from lighter data/self-registered data, respectively), 20,158/22,207 hand-to-mouth gestures (from hand IMU/proximity sensor, respectively) and 114,217/112,175 breaths (from the respiratory inductive plethysmograph (RIP)/bio-impedance sensor, respectively). The proposed system scored 8.3 ± 0.31 out of 10 on a post-study acceptability survey. The results suggest that PACT2.0 presents a reliable platform for studying of smoking behavior at the community level.

## 1. Introduction

The Center for Disease Control and Prevention (CDC) estimates that 20% of all deaths in the United States are related to tobacco consumption [1]. It is well established that cigarette smoking can cause diverse types of cancer, as well as heart, lung, and cerebrovascular diseases (among other diseases), resulting in 440,000 deaths annually [2]. Previous studies have shown that, annually out of billion worldwide smokers, between 30% and 50% of them attempt to quit smoking; however, approximately 70% of them fail to quit [3]. There is clear need for continued programmatic research on these lapses–relapses [4], the difficulties in quitting smoking, and on the development of intervention tools to support smokers in their attempts to maintain abstinence. One approach to support smokers as they quit smoking could be continuous reminders of (a) the frequency or pattern of their smoking; (b) measurements of daily nicotine consumption; and (c) the real-time impact of smoking on physiological variables such as heart rate or blood pressure, halitosis (bad breath) [5], etc. The first step toward the development of such methods is the ability to automatically detect smoking events in free-living smokers, which has always been elusive.

Traditionally, estimations of cigarette consumption have been collected through self-reporting methods such as logging in a paper or electronic diary, employing portable topography devices, and biomarkers etc. [6]. However, all of these methods tend to underestimate true cigarette consumption [7]. Diaries also do not provide sufficient information for evaluating health risks (such as total smoke exposure, maximum puff velocity, individual puff volume or duration, and post-puff respiratory events etc.) [2]. Recently, studies to develop sensors for the assessment of cigarette consumption have been conducted. The proposed sensors monitor one or several behavioral manifestations of smoking such as the lighting the cigarette, hand-to-mouth (cigarette-to-mouth) gestures, or characteristic breathing patterns during smoke inhalation. Because lighting a cigarette is an integral part of smoking, an instrumented lighter concept was introduced in [8] which tracked the lighter press and release events and recorded the frequency of cigarette consumption. Among commercially available e-lighters, Quitbit [9], is a special internet-enabled one to provide cigarette counts using integrated electronics in its heating coil.

Hand-to-mouth gestures during smoking have been detected by inertial sensors (accelerometer, gyroscope, and magnetometer) on the smoker's wrist [10,11], shoulder and arm [12]. These studies explored different Inertial Measurement Unit (IMU) placements on hand positions [10,13] or the minimum number of IMUs [11,12] necessary for accurate identification of smoking gestures. To recognize smoking from these IMU signals, multiple machine learning approaches have been proposed, such as random forest in [11,14], support vector machine (SVM) in [12], and hierarchical approaches in [15] etc. A radio frequency (RF) proximity sensor was employed in [16] to identify the cigarette-to-mouth gesture. Smoking has also been detected [17] through the analysis of subject's breathing patterns employing respiratory inductive plethysmograph (RIP) sensors [18]. Studies in [17,19–21] showed that the smoking has specific characteristic breathing patterns which can be easily identifiable. Authors in [22,23] employed two RIP breathing belts (abdominal and thoracic) and identified smoking patterns from these signals whereas the authors in [24] preferred single band RIP sensors. The classification algorithms used SVM [22,24] and hidden Markov models [23] for classifying the RIP signals. However, all of these mentioned sensors, individually, cannot truly ensure the non-reactive detection of smoking patterns [4]. The integration of multiple sensor systems into a single platform, as described in [25,26], allows for more accurate identification of smoking. Authors in [25] proposed an SVM-based approach, which combined wrist IMUs and a single band RIP sensor of the AutoSense sensor suit [27] and achieved a sensitivity of 96% for detecting smoking events in a controlled environment. These studies suggest that a combination of hand gesture and breathing may enhance the accuracy of smoking detection. However, this research contained no thorough systematic investigation of the association between smoking behaviors and smoking locations (i.e., home, office, free-living environment); or between smoking metrics and ambient factors or body motions (such as sedentary in vehicles, resting idly, slow or fast walking, etc.) which might further contribute to the accuracy of detection. In our earlier research [26] we proposed the Personalized Automated Cigarette Tracker (PACT), a system sensor employing a hand-to-mouth proximity sensor and an RIP breathing sensor. PACT demonstrated an F-score of 94% in the detection of smoke inhalations in a laboratory study [28–31]. Both SVM (in [28,29]) and decision tree ensemble (AdaBoost, bootstrap aggregating, random forests in [30,31]) approaches were explored on the sensor data of PACT system recorded in the controlled environment. However, PACT's accuracy was not thoroughly evaluated in naturalistic settings. Also, a limitation of the original PACT system was its large size and reliance on sensors with wired connections to an external data logger. To enable long-term free-living studies, it was necessary to improve the PACT system with miniaturization of the sensors and embedding of the data logging into the system.

The next generation of PACT (PACT2.0) described in this paper sets the following major goals: (1) implementation of a complete sensor suite for monitoring of all major behavioral manifestations of cigarette smoking (lighting events, hand-to-mouth gestures, and smoke inhalations); (2) miniaturization of the sensor hardware to enable its applicability for multi-day studies in naturalistic settings; and (3) introduction of new sensor modalities that might provide additional insight into smoking behavior e.g., body orientation, Global Positioning System (GPS), pedometer and Electrocardiogram (ECG) or provide an easy-to-use alternative (e.g., bio-impedance respiration sensor [32]) to traditional sensors; and (4) validation of the sensor suite in the community.

The PACT2.0 system consists of three custom-built devices: an instrumented lighter, hand module, and chest module with an embedded data log capability of more than two weeks. The instrumented lighter records the time and duration of all lighting events preceding smoking. The hand module integrates inertial sensors and an RF transmitter to track hand-to-mouth gestures. This module also includes a pedometer step counter. The chest module monitors breathing (smoke inhalation) patterns (inductive and bio-impedance respiratory sensors), cardiac activity (ECG sensor), chest movement (three-axis accelerometer), hand-to-mouth proximity (RF receiver), and captures geo-position of the subject (GPS receiver). A validation study (bench test, laboratory experiments, and human study on 40 subjects) is also presented in this paper to demonstrate the functionality of sensor modules and the acceptance of PACT2.0 system for long-term usage in naturalistic settings. The test results suggest that the proposed system represents a reliable platform for studying smoking behavior under free-living conditions.

This paper is organized as follows: Section 2 describes the wearable system, validation setup for the characterization of the sensors, and description of data collection; Section 3 shows the results obtained from the validation tests and data collection from subjects; and Sections 4 and 5 provide the discussion, future work and the conclusions of this study.

## 2. Methods

### 2.1. Wearable System

**Figure 1:**
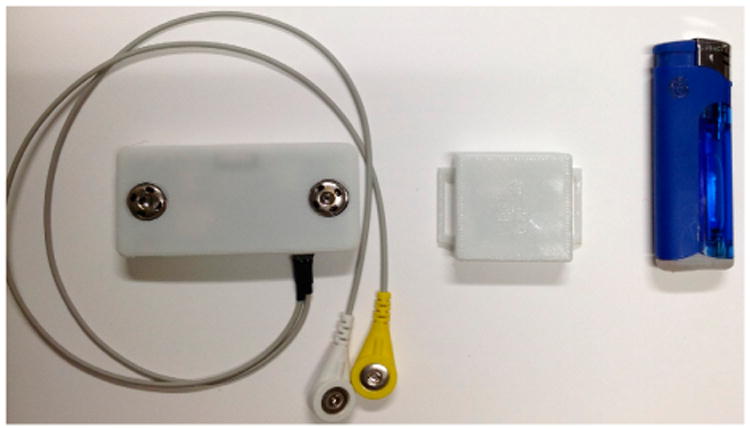
Chest module (**left**), hand module (**middle**), and instrumented lighter (**right**).

**Figure 2:**
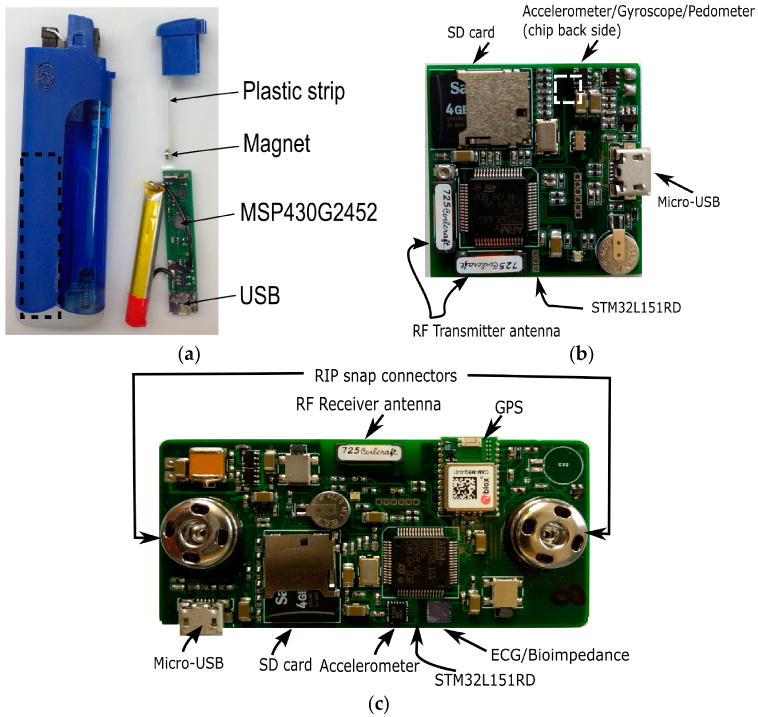
Components for: (**a**) instrumented lighter (**b**) hand module (**c**) chest module. Dashed-line rectangle in the lighter indicates where the board and battery are located inside the lighter.

The proposed multi-sensory wearable system was composed of three custom-built devices and an instrumented T-shirt. Figure 1 shows the custom-built devices: a commercially available lighter with an embedded small electronic board (38 × 8 × 1 mm), a hand module (35 × 35 × 10 mm), and a chest module (77 × 35 × 10 mm). Each module was equipped with a set of various sensors, a microcontroller, a storage unit, a USB communication unit, and a power source. Figure 2 shows the overall components for each module. The lighter used an MSP430G2452 microcontroller (Texas Instruments, Dallas, TX, USA), and the data was stored in a flash memory chip. The lighter was powered by a cylindrical Li-polymer battery of 3.7 V 210 mAh, and a micro-USB allowed for updating of the internal clock of the processor and access to the lighting records from the computer. The hand and chest modules used an STM32L151RD Cortex-M3 ARM processor (ST Microelectronics, Geneva, Switzerland) having 32 MHz CPU, 230 uA/MHz; a 4GB micro-SD card to store sensor data; a micro-USB interface to allow start/stop of data logging, retrieval of data stored in the SD card, updating of the internal clock of the processor, charging of the battery, and firmware upload. These were powered by a 3.7 V Li-polymer battery of 1000 mAh and 400 mAh, respectively. A detailed description of the sensing elements of each module is provided in Section 2.2.

**Figure 3:**
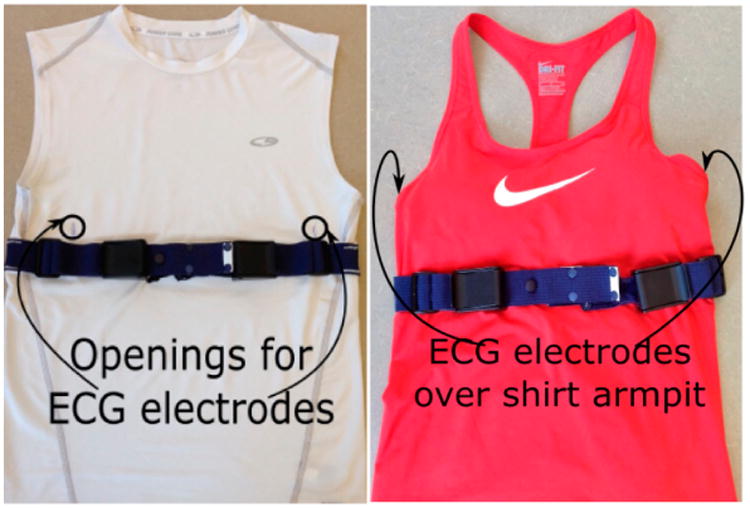
Instrumented T-shirt for male (**left**) and female user (**right**).

The proposed wearable system also integrated the chest module with a commercially available T-shirt with good recovery characteristics. The C9 by Champion® Power Compression (84% polyester/16% spandex) was used for male subjects and the Nike Women's Pro Cool Short Sleeve (84% polyester/16% spandex) for female subjects. A respiratory inductive plethysmograph (RIP) belt was sewn to the back of the T-shirt at the chest level; the length of the belt was adjusted through buckles located on the portion of the belt not sewn to the shirt. A comparative study in [33] showed that this configuration provided the best calibration stability for estimation of lung volume over a period of 24 h. Figure 3 shows the instrumented T-shirt (for both males and females) with the provision made for the placement of ECG electrodes (discussed in Section 2.2).

### 2.2. Sensing Elements

#### Instrumented Lighter

A commercially available lighter was modified to integrate the electronics to track the press and release events from the trigger through a Hall Effect sensor. The components of the instrumented lighter are shown in Figure 2. One end of a 36 × 3 mm plastic strip was glued to the trigger using an epoxy adhesive while the other end was glued to a magnet (D1007, diameter 3 mm and thickness 1 mm). A small electronic board (38 × 8 × 1 mm) contained the MSP430G2452 (Texas Instruments, Dallas, TX, USA) microcontroller, a USB port, and flash memory for data storage and battery. When the trigger is pressed, the magnet is in the proximity range of the onboard electromagnetic sensor, thus, the timestamp from the internal clock of the processor is read and stored in the memory. The timestamp is stored immediately after the trigger is released and the magnet moves out of the proximity range.

#### Hand Module

**Figure 4:**
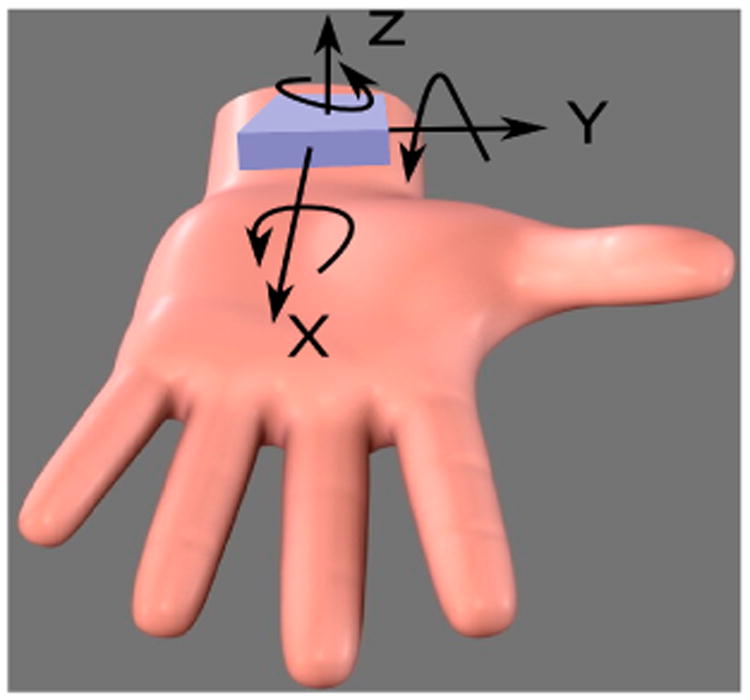
Accelerometer and gyroscope axes (positive direction) for the hand module.

The sensors in this module tracked hand movements and steps of a subject and provided a radio frequency (RF) transmitter circuitry of the hand-to-mouth proximity i.e., the proximity between the chest and hand modules. Hand motion was monitored using a 6-axis IMU (LSM6DS3, STMicroelectronics, Geneva, Switzerland) to capture the linear and angular velocity by the accelerometer and gyroscope, respectively. Figure 4 shows the accelerometer and gyroscope axes of the hand module; this same positioning was used regardless of the hand used for cigarette smoking. The accelerometer and gyroscope were configured to have a ±8 g and 2000 dps measurement range, respectively, with16bits of resolution. The IMU measurements were sampled by the microcontroller at a frequency of 100 Hz. The LSM6DS3 also includes a pedometer to count the steps of the subject. The pedometer was configured to count the total number of steps every 13.63s (the time needed to fill up the storage buffer of the processor).

**Figure 5:**
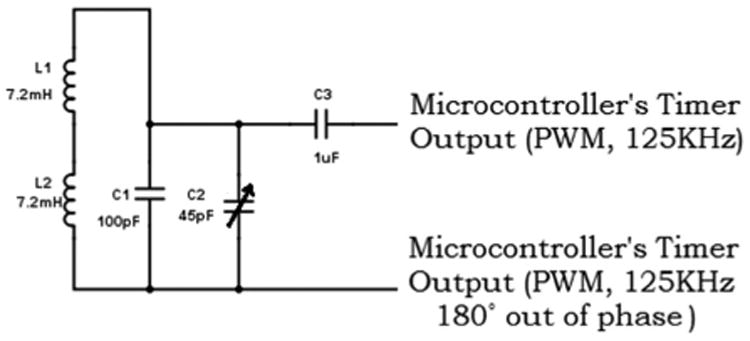
Radio Frequency (RF) transmitter circuit of the hand module.

For the hand RF transmitter (shown in Figure 5), the microcontroller was configured to generate two square signals of 125 kHz (2.8 V) using Pulse Width Modulation (PWM). These signals were connected to a tank circuit (consisting of L1–L2 and C1) and were set to be 180° out of phase to double the output signal at the transmitting antenna, ensuring maximum power. Here, the employed inductors (L1–L2) are Coilcraft 4513TC Radio Frequency Identification (RFID) transponder coils (miniature antennas of 11.5 × 3.5 × 2.5 mm, 7.2 ± %2 mH, 91 Ohm).

#### Chest Module

**Figure 6:**
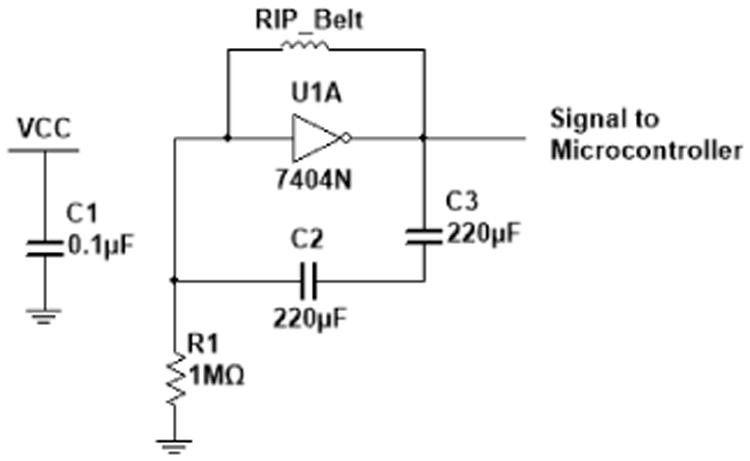
LC oscillator circuit employed for RIP sensing.

The sensors in the chest module monitored breathing patterns, heart activity, chest movement, hand-to-mouth proximity, and the geo-position of the subject. Breathing patterns were tracked by two technologies: (1) an RIP belt (SleepSense Inductive Plethysmography, S.L.P. Inc., St Charles, IL, USA); and (2) a bioimpedance sensor (ADS1292R, Texas Instruments, Dallas, TX, USA). The RIP belt captured the contraction and expansion of the chest and generated breathing waveform through an LC oscillator as shown in Figure 6. The resonant frequency of this oscillator is f_0_ = 1/(2π√(LC)), where L is the inductance of the belt (average inductance 2∼3 μH) proportional to the cross-section area of the belt's closed loop around the chest and C is the equivalent capacitance of the series capacitors C2 and C3. The microcontroller used a 32-MHz system clock, and following the Nyquist theorem, the system was limited to sample signals with resonant frequencies less than 16 MHz from the oscillator. Hence, capacitors C2 and C3 were set 220 pF to meet this criterion in which resonant frequencies were always under 16 MHz when the RIP belt was fully compressed and expanded. The microcontroller was configured to receive the pulses from the oscillator in one of the timer channel inputs and count the number of pulses that arrived over every 10 ms (100 Hz).

**Figure 7:**
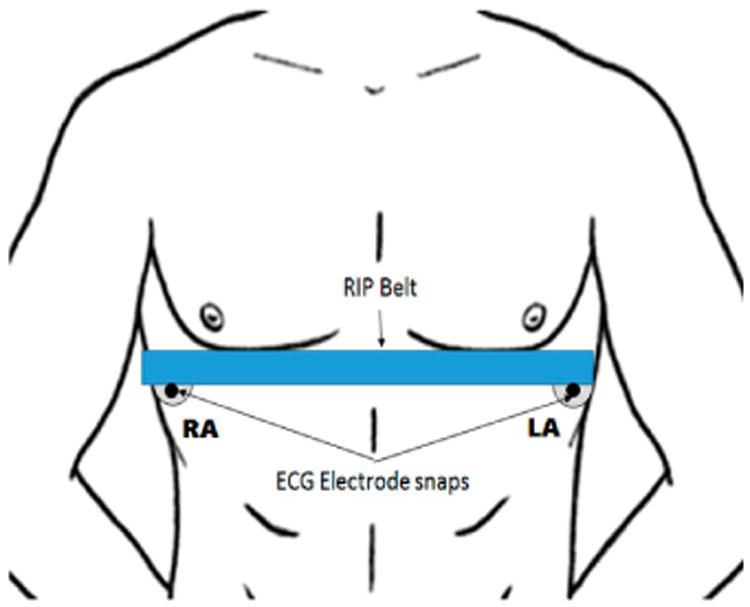
Electrode placement for the acquisition of respiration and heart activity (right arm: RA; left arm: LA).

The ADS1292R chip provided the capability to acquire both respiration and cardiac signals simultaneously through its internal bioimpedance and ECG sensors. An ECG Lead-I configuration was adopted for the data collection. The left arm (LA) and right arm (RA) electrode pair were placed on the side of thorax underneath the pectoral muscle and breast for male and female subjects, respectively. Figure 7 shows an example of electrode placement with the RIP belt for a male subject. Using the same electrode pair, a 32-KHz square wave was injected into the body, and the impedance across the electrodes captured the contraction and expansion of the thorax. The bioimpedance and ECG signals were amplified 3× and 12×, respectively. Finally, these resulting signals were sampled at a frequency of 1 kHz.

**Figure 8:**
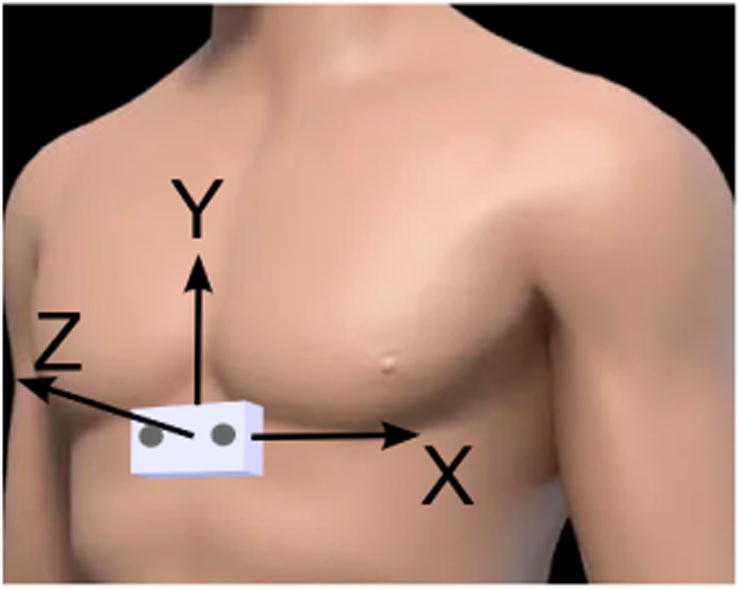
Accelerometer axes (positive direction) for chest module.

Tracking of the chest motion of the subject was done using a three-axis accelerometer (ADXL362, Analog Devices, Norwood, MA, USA) installed on the chest module. This accelerometer was configured to have a ±2 g measurement range with a resolution of 12 bits and an output data rate of 100 Hz. Figure 8 shows the accelerometer axes of the chest module.

The geographic position of a subject was tracked using a GPS receiver unit (Fastrax UC530), which records the location-coordinates from 12 GPS satellites. The GPS was configured in an intelligent power-saving mode (AlwaysLocate™) to adaptively adjust the navigation activity depending on the motions of the subject and surrounding environment [34].

**Figure 9:**
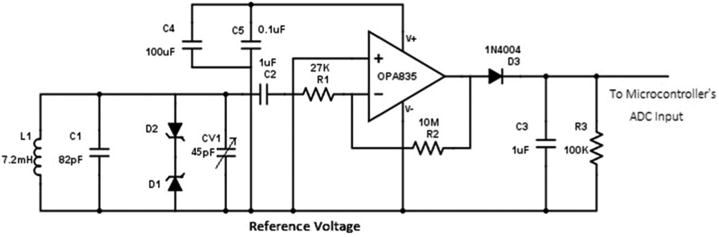
RF receiver circuit of the chest module.

Finally, an RF receiver circuit (Figure 9) was designed for the proximity sensor. A Coilcraft miniature antenna (the same as the transmitter module), was connected to capacitors (C1 and CV1) to form a tank circuit with a resonant frequency of 125 kHz. The output of the oscillator was filtered using an analog high-pass filter with the cutoff frequency of 6 Hz and rectified to generate a signal proportional to the strength of the RF signal. The circuit's output was fed to the microcontroller and digitized by an Analog to Digital Converter (ADC) with a resolution of 12 bits at a sampling frequency of 100 Hz.

**Figure 10:**
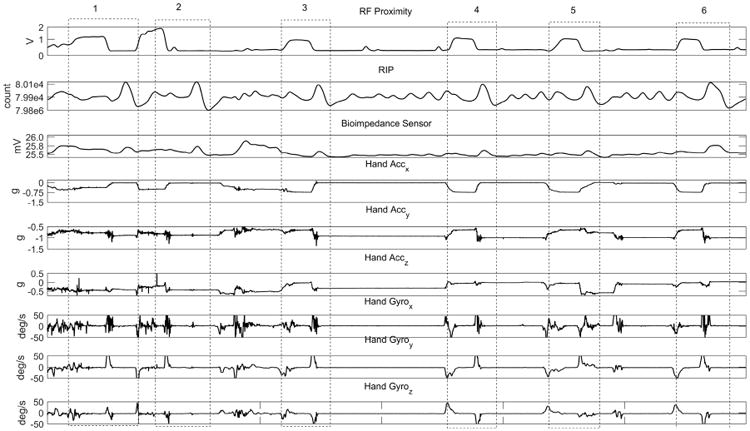
The responses of chest and hand sensors (RF proximity, RIP, bioimpedance, hand IMUs (Acc_x_, Acc_y_, Acc_z_ denote accelerometer X, Y, Z axis and Gyro_x_, Gyro_y_, Gyro_z_ denote Gyroscope X, Y, Z axis respectively) while a cigarette is being smoked using the dominant hand in a sitting posture. Six smoke inhalations are marked by dashed-line boxes from the manual video annotation.

An illustration of several sensor responses during a 2-min interval of cigarette smoking is shown in Figure 10. Using manual video annotation, six smoking events are marked in the figure by dashed boxes. The rise of the RF signal strength in the dashed box indicates the closeness of the wrist transmitter to the chest receiver module i.e., the hand-to-mouth proximity preceding cigarette puffs. Also, the hand IMU signals (Accelerometer marked as Acc_x_, Acc_y_, Acc_z_ and Gyroscope marked as Gyro_x_, Gyro_y_, Gyro_z_) suggest the presence of distinguishable patterns in those smoking events. The respiratory signals recorded by the RIP and bioimpedance signals also indicate characteristic breathing patterns specific to smoke inhalations.

### 2.3. Embedded Software

**Figure 11:**
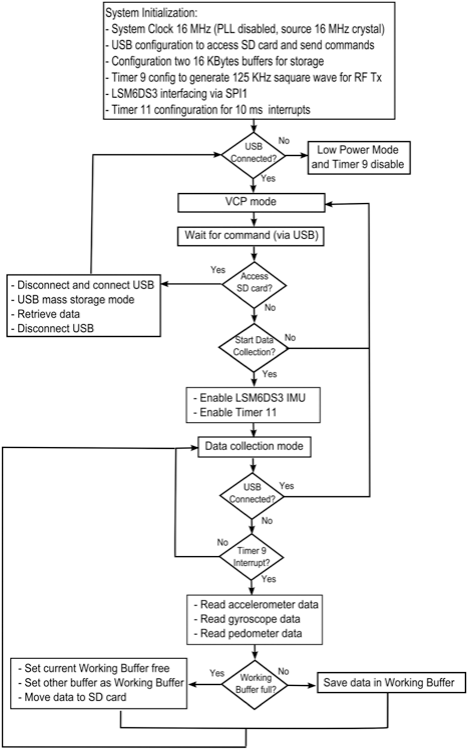
Hand module firmware.

**Figure 12:**
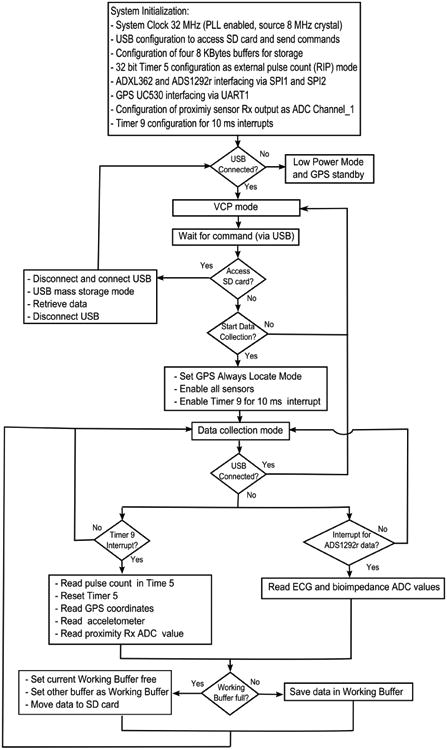
Chest module firmware.

The firmware for both hand and chest modules are shown in block diagrams in Figures 11 and 12, respectively). To limit the power consumption in the hand module, the system clock was set as 16 MHz, which is the minimum system clock required for this processor to support USB functionality. While for the chest module, the run mode clock was fixed as 32 MHz (highest system clock of the processor) considering the total number of sensors implemented in the chest module and meeting the Nyquist criteria for RIP pulse count (described in Section 2.2), the default USB interface was configured in a Virtual COM Port (VCP) mode, such that the processor waited for commands from the user to control the sensor activities, data retrieval (Mass Storage-MSC mode), or load new firmware (Device Firmware Upgrade-DFU mode). During data collection, a double buffer scheme was adopted to coordinate between reading the current sensor values and storing the previous ones in the binary file generated in the SD card with appropriate time stamps.

### 2.4. Time Synchronization and Drift Compensation

The PACT2.0 is a multi-sensor system with independent clocks in each of the devices. The time across devices needs to be synchronous over the duration of data collection. Slight differences in the clock frequencies and temperature-related variations in the frequency could lead to relative clock drift of up to several seconds per day. Therefore, the PACT system utilized two techniques to maintain the locks. First, the initial time synchronization between instrumented lighter, chest, and hand modules was established by sending computer time stamp (synchronized with an internet server) using a custom-developed LabVIEW application. Second, drift compensation was performed after completion of the data collection. The internal time stamps of the devices were read simultaneously (using same LabVIEW application) and the drift was corrected through a linear compensation as in Equation (1); where *t*, *t*_0_, *f*, and *t_c_* correspond to the raw timestamps, initial time of device set from the computer, correction factor, and compensated timestamps, respectively. The correction factor *f* was calculated as the ratio of (*t_device_* – *t*_0_)/(*t_pc_* – *t*_o_), where *t_device_* and *t_pc_* are the time of the device and computer time, respectively, at the moment of data extraction using the LabVIEW application.

(1) $$t_{C}=\frac{t-t_{0}}{f}+t_{0}$$

### 2.5. System Validation

#### 2.5.1. Bench Test

A series of bench tests were initially performed to assure the proper functionally of the system; these are described below.

##### (1) Idle test

For statistical characterization of the inherent noise of sensors in the hand and chest modules, both devices were set on a flat surface (undisturbed) with known location coordinates, and data were collected for 10 min. The mean and standard deviation of the noise measurements of the hand sensors (accelerometer, gyroscope, and pedometer) and chest sensors (respiration circuits, ECG, accelerometer, RF receiver, GPS) were computed. In the case of GPS, the difference between measurements (by the system) and the true coordinates were measured in distance using [35].

##### (2) Lighter test

The lighter was further tested to observe whether firstly it recorded all true lighting events and did not generate false lighting detections, and secondly to determine whether drift arose in its internal clock over the longer period of time. For this, the lighter was pressed 20 consecutive times a day (every half-hour) for a week; the trigger was kept pressed for a period of 5 s for each measurement to differentiate the release time. Manually counted lighting events were compared with total lighter press–releases recorded by the lighter. For drift analysis, root mean square error (RMSE) was used to compare the timestamps recorded and the true time stamps of these 168-h data.

##### (3) RIP characterization test

**Figure 13:**
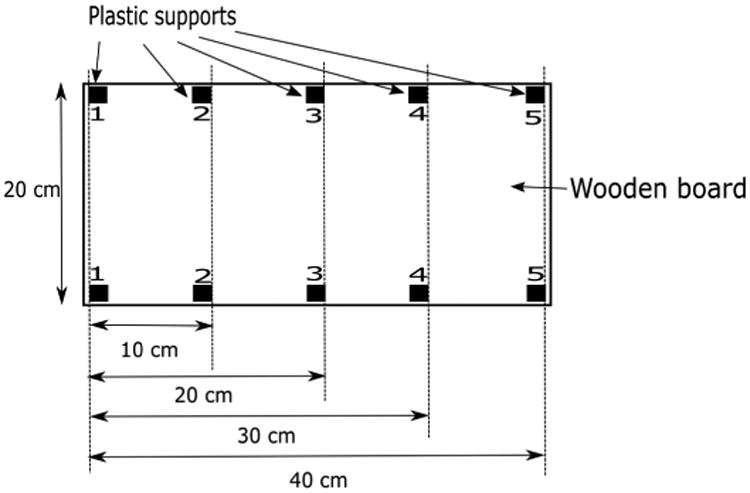
RIP noise characterizing test by simulating four chest sizes (60 cm, 80 cm, 100 cm, 120 cm).

Three additional tests were performed on an RIP sensor to verify its adaptability to all chest sizes; linearity in the belt expansion; and the potential effect of eddy current on the sensor. The first test consisted of breathless measurements, in which four chest circumference sizes (60 cm, 80 cm, 100 cm, 120 cm) [36] were simulated using the system depicted in Figure 13. This consisted of a wooden board with ten 5 × 3 cm 3D printed plastic supports installed at specific locations to establish perimeters according to the desired chest sizes. For the 60-cm test, the RIP belt was wrapped around supports 1 and 2. Subsequently, for the 80-, 100-, and 120-cm tests, the belt was placed between supports 1 and 3; 1 and 4; and 1 and 5, respectively. Three samples of 1 min duration were collected on each perimeter test and statistical measures such as mean pulse count, range, and standard deviation were computed. For the second test, the linearity response to a short displacement test was performed on the RIP belt. The belt was stretched 0 cm, 1 cm, 1.5 cm, 2 cm, and 2.5 cm. Data were recorded for 1 min to compute the mean pulse count for each measurement. The R^2^ measurement was used as the indicator of this linear response. The final test analyzed the impact of eddy current associated with the RIP belt. At high frequencies, the RIP belt may potentially induce eddy currents in the tissue. Hence, the RIP belt might respond to the proximity of other parts of the body (e.g., arm, hand) to itself. This impact was tested by placing a hand over the RIP belt for 2 min while worn, and the responses were statistically compared to the recording without the hand proximity.

##### (4) GPS receiver test

To validate the GPS navigation while in motion, a planned route was followed (∼1.57 km) while the sensor systems were worn by a research assistant. The route was split into four sections: two sections were walked, the third section was run, and, during the final section, the person remained stationary. The mean difference between the recorded (by the system) and the true coordinates were measured in distances [35].

##### (5) RF sensitivity test

The proximity sensor was further tested for potential electromagnetic interferences from the ambient sources and the sensor's maximum range of operation following the protocols used in PACT system [16]. Initially, the receiver circuit was carried in a research assistant's pocket (keeping the transmitter away) for 3 consecutive days while performing free-living activities [10]. The sensor signals were then analyzed for the deviation from the baseline that exceeds the threshold. Next, the greatest possible distance of the position of Tx–Rx pair was measured with the cigarette held in the mouth in all possible gestures [37]. Finally, a sensitivity test [16] was performed to verify whether this measured distance stayed within the range of operation of the Tx–Rx pair. For this test, the receiver was placed on a table and transmitter was moved away (perpendicularly) from the receiver. The received signal strength (in voltage) was plotted with respect to the distance between Tx–Rx pair to analyze the maximum range of operation of this sensor.

#### 2.5.2. Human Study

The final steps of validation were the collection of samples from regular smokers and the evaluation of the suitability of the system in naturalistic settings. The details are described below.

##### (1) Participants

Forty smokers with a history of smoking for at least 1 year and carbon monoxide (CO) levels >8 ppm were recruited through email announcements and fliers posted on the University of Alabama campus. These subjects consisted of 27 male and 13 female subjects (21 Caucasian, 12 Asian, 4 American Indian, 2 African American, 1 Hawaiian) with an average age of 25.25 ± 10.84 years (range: 19–62 years), average weight of 73.99 ± 17.64 Kg (range: 44–123 Kg), average Body mass index, BMI of 24.60 ± 6.12 kg/m^2^ (range: 18.21–45.88 kg/m^2^), average chest circumference of 85.63 ± 12.34 cm (range: 68–112 cm) and smoking history of 6.23 ± 7.25 years (range: 1–40 years). Of the 40 participants, 16 had a smoking history of 1–2 years, 10 had a history of 3–5 years, 8 had a history of 6–10 years, and 6 had a history of over 10 years. The self-reported cigarette consumption by the subjects was, on average, 11 ± 5.54 per day (range: 8–20) and with an average CO level of 14 ± 6.21 ppm (range: 8–33 ppm) measured in the screening process using a BreathCO vitalograph device [38]. Among the subjects, 16 reported smoking 5–7 cigarettes/day, 8 reported 8–10 cigarettes, 3 reported 11–13, 3 reported 14–17, and 10 reported 18–20 cigarettes/day. The study was approved by the Institutional Review Board (IRB) at the University of Alabama. Subjects reported that they were healthy and had no acute or chronic respiratory problems. Informed consent was received from all subjects. The participants received an $80 remuneration for participation in the study.

##### (2) Study Procedure

**Figure 14:**
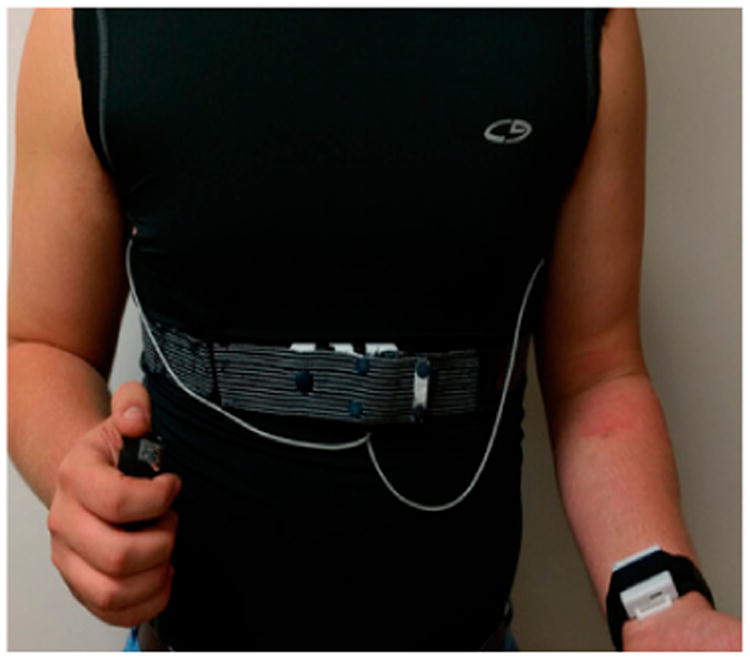
Personal Automatic Cigarette Tracker v2 (PACT2.0) applied on a male subject: chest module attached with the belt, hand module placed on the dominant hand of smoking and lighter on the non-dominant hand.

Data were collected from subjects in a controlled environment (∼3 h) at the University of Alabama followed by an uncontrolled study (∼24 h). Prior to starting the controlled study, subjects were instructed on how to self-apply the instrumented T-shirt and chest and hand modules. Several instrumented T-shirts (sizes: Male = S, M, L, XL, XXL and Female = XS, S, M, L, XL) paired with the RIP belts (51 cm, 81 cm, 110 cm, and 139 cm) were available to the subjects, who wore a T-shirt size one smaller than their regular size. After self-applying the system (shown in Figure 14), subjects performed two spirometry maneuvers: slow vital capacity (SVC) and forced vital capacity (FVC) [39] (each done three times) through a commercially available spirometer (Easy on-PC, ndd Medical Technologies, Inc., Andover, MA, USA). After the spirometry tests, subjects were asked to perform daily activities including smoking cigarettes: (1) reading aloud; (2) walking on a treadmill at self-selected slow pace; (3) walking on a treadmill at self-selected fast pace; (4) resting on a chair; (5) smoking while sitting on a chair; (6) talking on the phone; (7) eating in a cafeteria; (8) smoking while walking and talking; (9) smoking while standing and talking; (10) smoking while walking silently; and (11) smoking through a CreSS Pocket device (Borgwaldt KC, Richmond, VA, USA) while sitting. Activities had a maximum duration of 5 min, except for eating and smoking. Regarding the walking activities, the average slow and fast paces were 1.8 ± 0.3 mph and 3.0 ± 0.45 mph, respectively. Also, subjects carried and used the instrumented lighter to light their personal cigarettes during the controlled portion of the study. To facilitate annotation in the recorded sensor responses, the research assistant videotaped the entire session with an iON video camera time-synchronized with PACT sensors and marked the start and end timestamps of each activity in a smartphone application (aTimeLogger—Time Tracker [40]). Also, in this laboratory portion of the study, the finger-tip sensor of a carboxyhemoglobin monitor (Pulse CO-Oximeter Rad-57, Masimo, Irvine, CA, USA) was applied to the subject's non-dominant hand of smoking. The purpose of this commercial sensor was to validate the smoking activities from the SpCO saturation level i.e., changes in the blood CO as a result of cigarette smoking. Immediately after completion of the controlled study, the free-living portion began. Before leaving the laboratory, subjects were informed that there were no constraints on where to take the system and that they could perform any desired activity. They were instructed: (1) to remove the system only when taking a shower and self-apply again, (2) to use a similar customized smartphone application [40] to log the start and end timestamps of every cigarette they smoked, and (3) to return the system to the laboratory after 24 h of wear. The calibration of the sensor suite was performed twice (pre- and post- study) following the protocols mentioned in [33] to verify whether the sensors deteriorated in the longer run or not.

##### (3) Signal Processing

The signals acquired from the sensors for 40 subjects (both controlled and free-living) were needed for individual processing and to remove noise and motion artifacts. After analyzing the frequency content of the signals through Fast Fourier Transform (FFT) using a MATLAB script, first-order high-pass Butterworth filters with cutoff frequencies of 0.1 Hz, 0.001 Hz, and 1 Hz were implemented to remove the motion artifacts from RIP, bio-impedance, and ECG signals, respectively. Subsequently, for RIP and bio-impedance signals, an average Gaussian filter (of 10 and 100 points respectively) was used for smoothing. Finally, a notch filter was implemented to remove the 60-Hz power line interference from the ECG signal. Next, the IMU data was de-noised by a second-order low-pass Butterworth filter with a cutoff frequency of 2 Hz. Finally, for the proximity sensor signal, an average Gaussian filter of 50 points was used for smoothing.

##### (4) Data analysis

The quality of sensor signals (both controlled and free-living) was thoroughly investigated to determine whether the recorded data were usable for further analysis. Here the signals recorded within the study hour (between the start of the controlled portion and the end of free-living) were only considered. After plotting the sensor responses, the segments showing no valid signal (for example, lack of breathing signal due to the removal of PACT during showering, dislodged RIP belt, device malfunction etc.) were marked ‘unusable’.

Next, the following metrics were computed from the usable data (both controlled and free-living):

- the total number of lighter press–releases performed by all subjects;
- the total number of consumed cigarettes estimated from the lighter data. For this, the start and end times of the smoking session were extracted from the smartphone log. Using these timestamps, multiple (consecutive) lighter presses prior to lighting the cigarette were considered as one and the unexpected lighter presses during the non-smoking events were discarded;
- the total number of consumed cigarettes from the subjects' self-registration on their phone;
- the total number of hand-to-mouth gestures from the hand IMU data using the algorithm described in [25];
- the total number of breaths from the de-noised RIP and bioimpedance sensor using MATLAB's *findpeaks* algorithm (by finding local maxima);
- the total number of hand-to-mouth proximity movements from the RF sensor. The signal qualifies as a valid hand-to-mouth proximity movement if the amplitude > the threshold 70 mV (ten times the mean noise amplitude of 7 mV).
- the average heart rate during a silent smoking session (in a sitting posture) with the comparison of pre- and post-smoking heart rates. For this, a similar *findpeaks* algorithm was applied on the de-noised ECG signal to find the characteristic R-waves. The instantaneous heart rate (number of R-peaks per minute) was computed to average the heart rates during the sitting–smoking session, 5 min before smoking, and 15 min after smoking.

Finally, an analysis was performed to validate the inclusion of bio-impedance sensor as a potential alternative to RIP sensor. For this, the mean cross-correlation coefficient was computed between the bio-impedance and the SVC maneuvers for all subjects to determine the signal similarities. This value was also compared with the mean cross-correlation coefficient similarly computed between the RIP and SVC maneuvers.

##### (5) Survey on System Acceptance

Upon completion of the study, the subjects were asked to complete an ‘acceptability questionnaire’ to evaluate the acceptance of this system in natural conditions. Subjects were asked to rate the whole system (0—not comfortable at all, 10—very comfortable for daily use) in terms of comfort to wear for 24 h. It also contained the following questions: (1) whether the weight, size of the device and the electrodes placed on the body affected their freedom of movement; (2) whether the applied chest and hand module affected their pattern of smoking; and (3) whether they felt any pain while wearing the devices or noticed any post effects on their skin. Responses were analyzed to evaluate the tolerability of the system for the long-term application.

## 3. Results

### 3.1. Bench Test

From the idle test, the system noise measurements were obtained. Table 1 summarizes the results of the idle test.

From the lighter test, no false lighter press–release events were found in the lighter log, while 140 true press–releases were found over 168 h of testing. In the drift analysis, the RMSE value was calculated as 0.6708 s for the recorded timestamp of the lighter press vs. the true time stamp.

**Figure 15:**
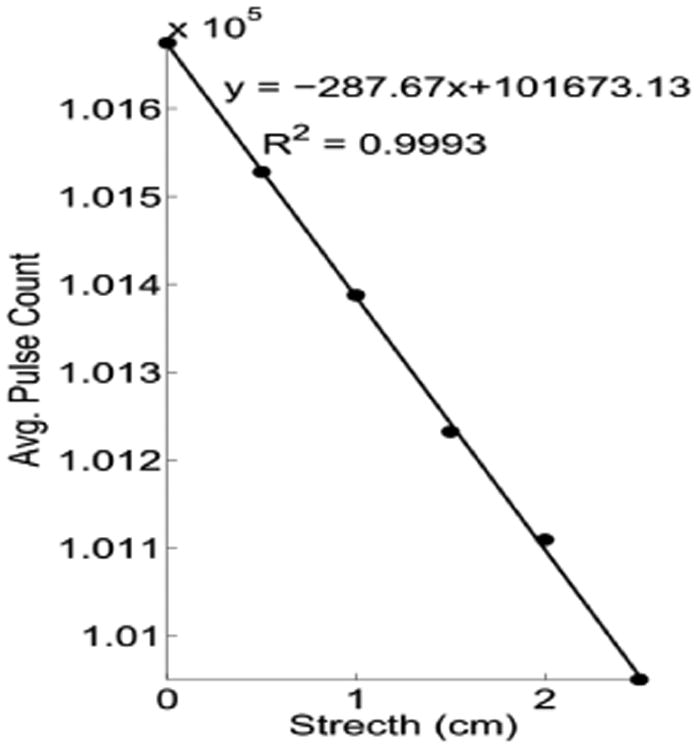
Results of the RIP linearity test with R^2^ statistics.

The results of the RIP bench testing for four simulated chest circumferences is provided in Table 2. Figure 15 shows the result of the linearity test with R^2^ parameter when stretching an RIP belt 0 cm, 1 cm, 1.5 cm, 2 cm, and 2.5 cm. The R^2^ value for this test was 0.9993. For the eddy current measurement, the mean pulse counts and the standard deviations for the portion of the signal with and without the presence of hand proximity were 86,527.69 ± 50.49 and 86,506.85 ± 36.79, respectively.

**Figure 16:**
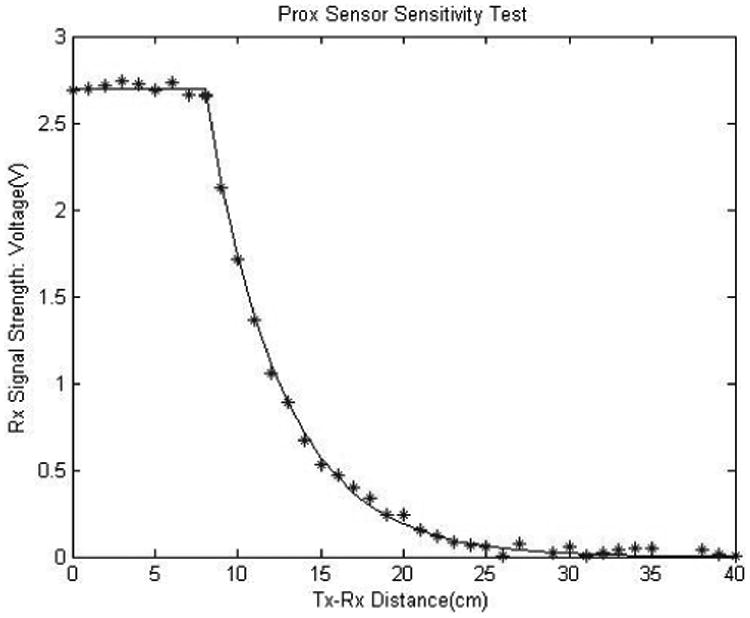
Proximity sensor sensitivity test. (Tx: Transmitter, Rx: Receiver, Prox: Proximity)

For the GPS receiver test, the average displacement of the recorded coordinates (by the system) from the true route was 24.53 ± 13.29 m. For the RF sensitivity test, in terms of electromagnetic interference, the receiver only showed 12 false positives in 72 h while carrying in the pocket and performing free-living activities. The receiver sensitivity curve is provided in Figure 16 with true data points and fitted curve. The maximum distance between the Tx–Rx pair in regular smoking gestures was ∼11 cm, which was found well inside the range of operation of this proximity sensor.

### 3.2. Human Study

Overall, the system recorded 943 h of data (during the study hours of the controlled and free-living portions) from 40 subjects. The data log system did not stop at any point of the study and did not fail to record any single sensor response. Hence, there was no data loss from the start of the protocol to the end of the free-living portion of the study. From the overall recording, 98.6% of data were found suitable for computer analysis, while the rest were unusable (1.1% owing to sensor system removal and 0.3% for ambiguous sensor response). Table 3 summarizes the recorded information from these data.

Regarding the instrumented lighter, eight false lighting events were found during the non-smoking activities of protocol section. In the free-living portion of the evaluation, the number of false lighting events during non-smoking activities was 19. The total number of consumed cigarettes estimated from the lighter records was 337, whereas the subjects reported 319 cigarettes in their smartphone application. By comparing the subjects' individual self-report and the lighter event log, it was estimated that 34 cigarettes were not reported by the subjects. Five subjects reported multiple cigarettes as one smoking event. It was estimated that 16 cigarettes might have lit by a lighter other than the instrumented lighter.

From the ECG data of a sitting-silent-smoking session of all subjects, the average heart rate was 112.03 ± 9.56 bpm (beats per minute) with 81.54 ± 7.28 bpm in pre-smoking and 89.69 ± 10.78 bpm during the post-smoking session.

From the spirometer SVC test data, the mean cross-correlation coefficient was 0.5438 between the bio-impedance and spirometer signal and 0.6275 between the RIP and spirometer.

In terms of acceptance, the subjects scored an average of 8.3 ± 0.31 out of 10 in the acceptability questionnaire. In the survey, they also reported that the devices' weight, size or electrodes did not affect their freedom of movement or pattern of smoking. They also did not feel any pain while wearing the devices or any noticeable post effects on their skin. The chest and hand modules did not get dislodged nor did they slip at any point during the study, and the tension of RIP belt was maintained throughout.

## 4. Discussion

The sensor modules of the PACT2.0 system produced accurate responses in all circumstances (different body motions, gestures, surrounding environments, etc.), thereby achieving one of the prime goals of this study. Also, the instrumented lighter reliably recorded all lighting events with accurate timestamps. The initial bench tests also demonstrated that the negligible amounts of noise present in the sensor modules were not large enough to impact the overall performances of the sensors. From the RIP linearity test, changes in the breathing belt characteristics were found to be uniform across all magnitudes of contraction and expansion. The RIP bench test and the human study on adult subjects with chest circumferences ranging from 68 to 112 cm confirmed that the system was capable of accommodating a variety of chest sizes. In the laboratory test, there was no indication of abrupt changes in response when other parts of the body came in close proximity to the sensors, confirming the limited impact of eddy currents. These results validate the suitability of the high-frequency RIP sensor module of the system.

The GPS receiver was reasonably accurate in terms of GPS noise (average displacement of 15.11 m in static conditions, 24.53 m in dynamic conditions). However, one known limitation is that the GPS used in PACT2.0 is unable to receive the location coordinates from the satellite when it is inside a building that blocks the RF signal from GPS satellites. The system continues logging its last received geo-coordinates before entering the building until a new coordinate is available. However, the place where people smoke (whether in the home, office or at social gatherings) can still be extracted from the recorded coordinates. This GPS navigation (outdoor) combined with the chest acceleration and step counts can also be used to identify movement status of the smoker during smoking and might contribute to improving the accuracy of detection algorithms.

The placement of the wristband containing the hand module was experimentally located on the upper wrist position as close to the elbow as possible to confirm the better reception of the RF signal during smoking events. For a typical cigarette-to-mouth gesture, the transmitter was previously recorded at a distance of 10–15 cm from the receiver in the PACT system [16], which agrees with the range found for PACT2.0 (11 cm). Also, the noise level in the RF receiver module (∼7.31 mV found from the ADC data and in digital oscilloscope) can be ignored considering the full range of signal amplitude (2.8 V). Similar to PACT, the RF sensor of the PACT2.0 system had negligible electromagnetic interference (12 false positives in 36 h of daily usage).

The ECG frequency spectrum is generally between 0.05 Hz and 150 Hz. To avoid the aliasing effects and the high-frequency spikes, a high ADC sampling frequency (1 kHz) was set to measure the heart rate from these signals, following the research in [41]. To capture the respiration signals using the same pair of electrodes, the thorax area was selected for the placement of ECG electrodes instead of the standard chest region. The wave shape, amplitude, and frequency of the ECG waveforms recorded from the electrodes placed on the thorax area were found to be similar to those obtained from the chest area (not shown here). These findings were highly consistent with the results of research in [42].

The validation of human study data was mainly accomplished by identifying distinguishable signal features of individual sensor modules. The breathing waveforms of the major breathing conditions: breathing with/without speech (sitting quietly and reading aloud), labored breathing (walking on a treadmill), and breathing during activities like eating and smoking etc., were distinguished with signals from both RIP and bioimpedance sensors.

From the study data (controlled and free-living), total breath counts by the bio-impedance sensor (112,175) were found to be comparable to those obtained by the RIP sensor (114,217) with an overall variation of 1.78%. Also, the similarity in cross-correlation values (0.6275 vs. 0.5438, respectively) with the spirometer signal demonstrates that the bio-impedance sensor has the potential to be a reliable alternative to RIP sensor, as the latter has the limitation of clothing integration, washing, and potential impact of motion artifacts [31]. The responses of the bio-impedance sensor (plotted in Figure 11), analogous to RIP signals, suggest the presence of distinguishable patterns during smoking puffs. Here the application of a computer algorithm on bio-impedance signals for automatic recognition of smoking patterns is likely to be informative and of great utility, both in terms of basic research on smoking as well as in the use of this system for clinical smoking interventions.

The ECG was consistently recorded across various activities. During the sitting silent–smoking session, heart rate increased significantly during smoking relative to the pre-smoking resting state and returned to baseline levels within 15 min of smoking, which is consistent with the findings in [43].

The hand IMUs and proximity sensor registered different types of hand-to-mouth gestures during eating, talking on a cell phone placed at an ear position, and smoking in different postures (sitting, walking, standing etc.).

The instrumented lighter registered 337 cigarettes during the free-living portion of the study, while 319 smoking events were self-reported. The source of discrepancy may be both in false positives generated by the lighter and false negatives from self-reporting. This information could be further validated by analyzing other sensor responses. A number of false lighting events were observed even in non-smoking activities. From the video recording, these false lighting events during the controlled portion of the study were identified as ‘user errors’ and hence ignored. As no video recording was involved in the free-living portion, the analysis of other sensor signals might help characterize the occurrence of these lighting events in non-smoking activities. This analysis is left open as a future work.

The comfort and the subjects' acceptance of the long-term usage were assessed by the ‘acceptability questionnaire’ survey. All 40 subjects confirmed that the PACT2.0 system was comfortable enough for wearing for a full day. Moreover, the instrumented t-shirt can be reused after machine washes. Given this feature, the system might be employed continuously for weeks.

The human study not only validates the sensor responses but also substantiates the robust data-logging capability of the system. With suitable SD cards, the system can record sensor data for months. However, the battery will demand regular recharging every 48 h. This can be solved by incorporating bigger batteries of higher capacity, but this might make the system heavier to wear. The proposed system consumes ∼33 mA in run mode and ∼8 uA in low-power mode.

The proposed PACT2.0 is an improvement on the sensor systems previously reported in the literature, including the original PACT system which was the first fusion approach in smoking research employing an RIP breathing sensor to recognize specific breathing patterns of smoking and a proximity sensor to detect hand-to-mouth gestures. For wear convenience, PACT2.0 relies on a single thoracic RIP belt and a miniaturized version of the proximity sensor. The original PACT system had a Tx antenna of 40 × 15 × 5 mm dimensions and an Rx antenna of 100 × 110 × 5 mm dimensions, while the PACT2.0 has 11.5 × 3.5 × 2.5 mm antennas at both the Tx and Rx end. Also, the Rx antenna was integrated into the chest module of the PACT2.0 system, excluding the necessity of additional clothing for the PACT system. Other two-sensor modalities previously used in smoking research such as hand IMUs and instrumented lighters are included in PACT2.0. PACT2.0 also facilitates the opportunity to explore new instrumentations that have not been previously used in smoking studies, such as the bio-impedance respiration sensor that may be more convenient to use than the RIP sensor, the ECG sensor to monitor instantaneous changes of heart rate while smoking, body orientation sensors such as chest IMU and pedometer, and GPS etc. Also, the validation study of PACT2.0 was performed in the largest study to date. The generated dataset would provide the possibility to analyze the real-life smoking behaviors. For example, a body orientation sensor will inform on how often people smoke while lying down. The GPS will provide information about smoking locations (common places like home or office) and if locations may have any impact on smoking habits etc. The physiological sensors of PACT2.0 (bioimpedance respiration sensor and ECG) are anticipated to enable the monitoring of smoking from the instantaneous changes of physiological parameters occurring during smoking.

This manuscript focuses on the electronics and instrumentation of PACT2.0. The signals collected in the validation study will also be further analyzed to recognize individual smoking events, characterize metrics of smoke exposure, etc. The pattern recognition and classification methods previously reported [28–31] will be adapted to be used with the instrumentation of PACT2.0. Additional methods of signal processing and pattern recognition methods for newly included sensors (ECG, bioimpedance, GPS, body orientation sensors etc.) will be developed and reported in the future.

In summary, PACT2.0, in its current version, is readily available for use in multi-day research studies of cigarette smoking. Since PACT2.0 requires an instrumented garment, a hand sensor, and the instrumented lighter, it might not be feasible for everyday use at the present time. However, with the development of smart textiles, the garment-integrated sensors may become suitable for everyday use, thus enabling the application of PACT in everyday monitoring and behavioral smoking cessation interventions. Another limitation is that PACT2.0 does not yet facilitate any real-time alerts, feedback, or interactions with the user about their smoking inhalations and patterns of smoking. In this current version, the data logged on the SD card can only be processed offline, and the user needs to start/stop data logging sessions manually. The next step of the ongoing research could be the inclusion of a low-power Bluetooth module and the development of a dedicated smartphone application for remote configuration of sensor parameters, control of data acquisition sessions, and real-time interaction with the users.

## 5. Conclusions

PACT2.0 is a robust multi-sensor platform enabling state-of-art research of smoking behavior in the community. It is envisioned here that the proposed multi-sensor combination will be an effective solution for objective monitoring of smoking patterns in the wild and provide a platform for sensor-driven behavioral interventions.

## Figures and Tables

**Table 1:** Idle test noise characterization results.

Module	Sensing Element	Noise Mean Value	Noise Standard Deviation
Hand	Hand Accelerometer	0.19 g	0.00324 g
	Hand Gyroscope	10 dps	1.57 dps
	Pedometer Step-Counter	0 count	0 count

Chest	Chest Accelerometer	0.04 g	0.00416 g
	respiratory inductive plethysmograph (RIP) Sensor	5.23 count	1.29 count
	Bioimpedance Sensor	0 mV	0 mV
	ECG (Electrocardiogram) Sensor	0.007 mV	0.003 mV
	Proximity Sensor	7.31 mV	1.21 mV
	GPS (coordinate displacement)	15.11 m	2.639 m

**Table 2:** Results of chest module noise tests for RIP.

Simulated Chest Circumference	Average Mean Pulse Count	Average Pulse Count Range	Average frequency (f_0_) Measured (MHz)
60 cm	102,376.76 ± 1.55	6.33 ± 1.15	10.23
80 cm	96,199.76 ± 0.74	5.67 ± 4.73	9.61
100 cm	92,954.09 ± 1.75	7.67 ± 4.16	9.29
120 cm	89,088.46 ± 1.31	5.67 ± 2.89	8.90

**Table 3:** Recorded information from the study data of 40 subjects.

Recorded Events	Controlled Portion	Free Living Portion	Total
Lighter press–release	193	356	549
Cigarette consumption estimated from lighter data	185	337	522
Cigarette consumption from self-report	185	319	504
Hand-to-mouth gestures from IMUs	2519	17,639	20,158
Breaths from RIP sensor	14,232	99,985	114,217
Breaths from bio-impedance	13,629	98,546	112,175
Hand-to-mouth proximity	2819	19,388	22,207
